# The Relationship between Addictive Use of Short-Video Platforms and Marital Satisfaction in Older Chinese Couples: An Asymmetrical Dyadic Process

**DOI:** 10.3390/bs14050364

**Published:** 2024-04-25

**Authors:** Jinsong Deng, Menmen Wang, Weiqi Mu, Siying Li, Ninghao Zhu, Xiong Luo, Lan Yi, Yahan Wu, Kexin Wang, Mingjie Zhou

**Affiliations:** 1 CAS Key Laboratory of Mental Health, Institute of Psychology, Chinese Academy of Sciences, Beijing 100101, China; jace404@outlook.com (J.D.); muwq@psych.ac.cn (W.M.); lisy@psych.ac.cn (S.L.); zhunh@psych.ac.cn (N.Z.); luox@psych.ac.cn (X.L.); yil@psych.ac.cn (L.Y.); wuyh@psych.ac.cn (Y.W.); 2 Department of Psychology, University of Chinese Academy of Sciences, Beijing 100049, China; 3 College of Media and International Culture, Zhejiang University, Hangzhou 310058, China; wangmenmen@zju.edu.cn

**Keywords:** addictive media use, short-video platforms, older couple, marital satisfaction, APIM (actor-partner interdependence model)

## Abstract

Increasing evidence indicates that the addictive use of social media can have a detrimental effect on marital satisfaction, due mainly to the decrease in time and focus given to one’s spouse. However, the impact of social media use among older couples remains under-investigated, and the research that does exist relies on individual-level data that do not allow the exploration of the dynamics between the dyadic partners. Therefore, the present study focused on older adults’ use of short-video platforms, as these have been shown to be particularly addictive for older adults. A sample of 264 older couples was gathered (mean_age_ = 68.02, *SD* = 8.68), and both spouses completed surveys reporting addictive use of short-video platforms, negative emotions, and marital satisfaction. Using an actor–partner interdependence model, we found an asymmetrical dyadic process in that the addictive use of short-video platforms by the wives was not only related to their own negative emotions, but also those of their spouse, as well as to decreased marital satisfaction. Meanwhile, addictive use by the husbands seemed to relate only to their own increased negative emotions, as well as to decreased marital satisfaction. Together, the findings from this study reveal dyadic dynamics with delineated pathways through which the addictive use of short-video platforms can damage older couples’ interactive processes and marital satisfaction.

## 1. Introduction

A short-video platform (SVP) is a social media format that has grown popular during the Web 2.0 period and allows users to record their own lives and create videos ranging from a few seconds to a few minutes in length, and to browse and like others’ videos [1]. According to the China Internet Network Information Center’s 2023 Statistical Report, the total number of SVP users had already exceeded 1 billion by the end of June, accounting for 95.2% of all Internet users [2]. Notably, in China, the usage rate of SVPs among older adults exceeds 80% [3].

While older adults can benefit greatly from SVP, as they offer access to information and entertainment, overuse can lead to SVP addiction and to marital dissatisfaction. In fact, addiction is a prominent issue with SVP use [4,5]. Addictive use of (social) media exists across numerous online platforms, including the Internet in general [6], Facebook [7], and Instagram [8], and is related to a decline in relationship quality in both dating and married couples. We believe that, in the Chinese context, the association between addictive social media use and relationship quality may be more pronounced in older married couples than in those in early or middle adulthood. With age, older couples experience greater developmental changes, as their social contacts dwindle, making them more dependent on their spouse for companionship and emotional support [9]. Thus, the disruption and interference from SVP in older couples’ usual interactions may be more salient.

With the rising popularity of SVP among older adults in China and the established negative impact of the addictive use of social media on the quality of married couples’ relationships, we inferred that there could be a connection between the addictive use of SVPs and the marital satisfaction of older couples. Therefore, the current study investigated the predictive role of the addictive use of SVP in marital satisfaction among older Chinese couples and further explored the potential mediation effect of one’s negative emotions.

The research on the effects of the addictive use of social media on romantic relationships is growing, yet few studies have employed dyadic data to explore relationship dynamics. Researchers have thus called for studies in which participants are paired up with their partners as a way that explores both the actor effect (e.g., whether a husband’s addictive social media use reduces his marital satisfaction) and the partner effect (e.g., whether a husband’s addictive social media use reduces his wife’s marital satisfaction) [10]. Men and women may also differ in how they perceive their spouse’s problematic media use. McDaniel et al. found that wives reported their husbands’ social media use as being more problematic than husbands did regarding their wives [11], while Booth et al. suggested the opposite: that husbands were more sensitive to their wives’ problematic media use than wives were of their husbands’ social media use [12]. These conflicting findings demonstrate that further research is necessary to better understand the gender difference in perceived problematic media use in marriage.

Given the importance of dyadic reactions and evaluations within a marriage, as well as the potential for gender differences, an interdependent and integrated framework is essential to investigate the mutual influence of two spouses’ media use behaviors upon one another. The present study therefore expanded upon prior research by utilizing an actor–partner interdependence model (APIM) to examine gender differences and mutual influences, as well as the mediation process via indirect pathways.

## 2. Theoretical Foundations and Hypotheses

### 2.1. Actor Effect of Addictive SVP Use on Marital Satisfaction: Displacement Theory and Self-Perception Theory

The influence of users’ addictive SVP use on their marital satisfaction can be strongly supported by displacement theory, which posits that time is not elastic [13,14] and, as such, people who spend excessive amounts of time on media may not then have enough time to foster intimacy in their face-to-face relationships. Those who are addicted to media in particular may be less likely to feel satisfied about their marriage because the beneficial interactions with their partner have been drastically replaced by their media engagement. This negative association between addictive SVP use and marital satisfaction can also be explained by self-perception theory [15], which suggests that people draw conclusions in their attitudes and beliefs based on their own behavior as though they were an observer. For example, when someone opts to dedicate their time to media activities instead of to their partner, they may infer that their feelings for their partner are not as strong and become less likely to put effort into the relationship. Research has supported this interpretation by documenting the detrimental consequences of addictive (social) media use in romantic relationships. For instance, Bouffard et al. revealed that the more one uses Instagram, the less satisfied they are with their relationships [8]. Meanwhile, Abbasi et al. found that Facebook addiction is associated with increased marital dissatisfaction [7].

Several studies have attempted to identify the potential mechanisms at play when addicted media users experience reduced relationship quality. Gull et al. revealed that excessive social media use generates feelings of loneliness and experience a lack of companionship in romantic relationships, which drastically reduces their satisfaction [16]. Aside from lessening one’s interpersonal intimacy, media addiction may also cause emotional issues. Studies have shown that addictive (social) media use can adversely affect an individual’s emotional wellbeing, leading to heightened anxiety and decreased self-esteem [17]. This can negatively impact one’s emotional happiness and thereby lead to dissatisfaction in their relationships.

Considering these previous findings, we hypothesized the following:

**H1.**  *Addictive SVP use in older adults is negatively related to their marital satisfaction (i.e., actor direct effect).*

**H2.**  *Addictive SVP use in older adults is positively related to their own negative emotions and then adversely to their own marital satisfaction (i.e., actor indirect effect).*

### 2.2. Partner Effect of Addictive SVP Use on Marital Satisfaction: Social Exchange Theory

The impact of addictive SVP use on a spouse’s marital satisfaction can be explained by social exchange theory. This theory suggests that partners continually assess the costs and rewards of their current relationship, trying to satisfy their needs by adapting or making changes while minimizing their costs with their partner [18]. When one perceives the interspousal exchange as being unbalanced, such as when one spouse devotes more energy to the relationship but receives less in return from their partner (e.g., reduced attention, less emotional support, or fewer meaningful interactions), this will have deleterious impacts on the relationship [19]. To maintain a harmonious marriage, it is essential that both spouses provide ongoing validation and care to one another [20]. Individuals must feel confident that their spouse will be attentive to their needs, regardless of circumstances or time. However, a common experience in romantic interactions in today’s media-saturated world is of one constantly checking their phone while their partner tries to maintain their attention. When an individual is addicted to media, they may be less attentive to their partner’s needs. Furthermore, considering the characteristics of addictive behavior [21], older individuals who are addicted to SVPs may experience greater conflict with their spouse or neglect important joint activities while they are engaged in media consumption, thus triggering dissatisfaction from their spouses. In short, the more time and energy one spends on media, the more likely it is that their spouse will feel hurt and disregarded [22].

Research has further supported the negative impact of one’s excessive (addictive) media use on their spouse’s relationship satisfaction. For instance, Morgan et al. revealed that respondents experienced feelings of distraction from the present, as well as relational dissatisfaction due to their romantic partners’ addictive media use [23]. Additionally, Booth et al. indicated that problematic media use is related to a spouse’s lower responsiveness in a relationship because they feel they are already being snubbed by their distracted partner, thus leading to lower relationship outcomes [12].

When one spends too much time viewing short videos or interacting with other SVP users on the platform, and not enough time engaging with their partner, this can cause the spouse to feel neglected and excluded (i.e., reduced rewards), which can then result in an overall decrease in satisfaction in the relationship [24]. Meanwhile, being snubbed by the addicted spouse may trigger more conflict, as research has found that greater amounts of media interruption or interference are associated with increased fights and poorer communication [11,25,26,27]. Blieszner has noted that older individuals experience a decrease in their social contacts, and, in response, they become more reliant on their spouse for companionship and emotional support [28]. Considering this, we speculated that older adults would experience negative emotions and decreased marriage satisfaction in response to their spouse’s addictive SVP use. Furthermore, considering the existing theoretical reasoning and empirical evidence regarding the actor–direct effect, we expanded our inference that addictive SVP use by one partner will trigger negative emotions in both partners, then decrease both partners’ marital satisfaction. As such, we proposed the following hypotheses:

**H3.**  *Addictive SVP use in older adults is negatively related to their spouse’s marital satisfaction (i.e., partner direct effect).*

**H4a.**  *Addictive SVP use in older adults is positively related to their spouse’s negative emotions, and then adversely to their spouse’s marital satisfaction (i.e., partner–actor indirect effect).*

**H4b.**  *Addictive SVP use in older adults is positively related to their own negative emotions, and then adversely to their spouse’s marital satisfaction (i.e., actor–partner indirect effect).*

**H4c.**  *Addictive SVP use in older adults is positively related to their spouse’s negative emotions, then adversely to their own marital satisfaction (i.e., partner–partner indirect effect).*

## 3. Materials and Methods

### 3.1. Participants and Procedure

A cross-sectional questionnaire-based survey was conducted in Sichuan province, China, in May and June 2023. Door-to-door visits were conducted across the community to invite residents to participate in the survey. The eligibility criteria included only older couples where both spouses were aged 55 and above [29] and were currently married. Participants were explicitly informed about the confidentiality and voluntary nature of their participation, and they were given the option to withdraw at any time. The questionnaire was distributed in an electronic format, ensuring that responses would be submitted without any values missing. The present study involved 264 heterosexual couples from China, with the average age of participants being 68.15 years (*SD* = 8.58, age range of 55 to 96 years). With regard to their education, 7.0% of participants had completed education below primary-school level, 27.7% had attained an elementary-school level education, 35.2% graduated from junior high school, 19.7% completed high school or vocational school, and 10.4% completed college-level or higher education.

### 3.2. Measures

#### 3.2.1. Demographics

Demographic variables included age and gender. Considering the influence of educational level on individuals’ marital satisfaction in previous studies [30], we also assessed the highest level of personal education achieved by respondents.

#### 3.2.2. Addictive SVP Use

The six-item Short-Form Video Application Addiction questionnaire [5] was used to assess respondents’ addictive SVP use, with sample items including “This short-form video app interferes with doing social activities” and “I feel anxious if I cannot access this short-form video app”. Respondents rate each item using a scale ranging from 1 (strongly disagree) to 7 (strongly agree). The score is generated by calculating the mean of the six items, with a higher score indicating a more severe level of addictive SVP use. In the current study, Cronbach’s alpha coefficient of this scale was 0.909 for the husbands and 0.874 for the wives.

#### 3.2.3. Marital Satisfaction

The marital satisfaction subscale of the Evaluation and Nurturing Relationship Issues, Communication and Happiness scale (ENRICH) [31] consists of 10 items used to measure marital satisfaction or dissatisfaction with one’s partner. Sample items include “I do not like my spouse’s personality and habits” and “I am satisfied with the emotional expression between us”. Each item is rated using a scale ranging from 1 (strongly disagree) to 5 (strongly agree). The mean of the 10 items is calculated to assess the respondent’s marital satisfaction, where higher scores indicate greater levels of satisfaction in their marriage. Previous research conducted with couples from mainland China has validated the original structure of the marital satisfaction subscale of the ENRICH within the Chinese language and cultural context [32]. Cronbach’s alpha coefficient for this scale in the current study was 0.954 for the husbands and 0.961 for the wives.

#### 3.2.4. Negative Emotions

A list of five adjectives describing negative emotions was provided to participants who were asked to evaluate the extent of their experience with these emotions during the previous week (e.g., “upset”, “hostile”) [33]. They responded using a scale ranging from 1 (never) to 7 (always), with higher scores reflecting greater levels of negative emotions. The negative emotions score was obtained by calculating the mean of the ratings for the five negative adjectives. In this study, Cronbach’s alpha coefficient was 0.909 for the husbands, and 0.902 for the wives.

### 3.3. Data Analyses

**Figure 1:**
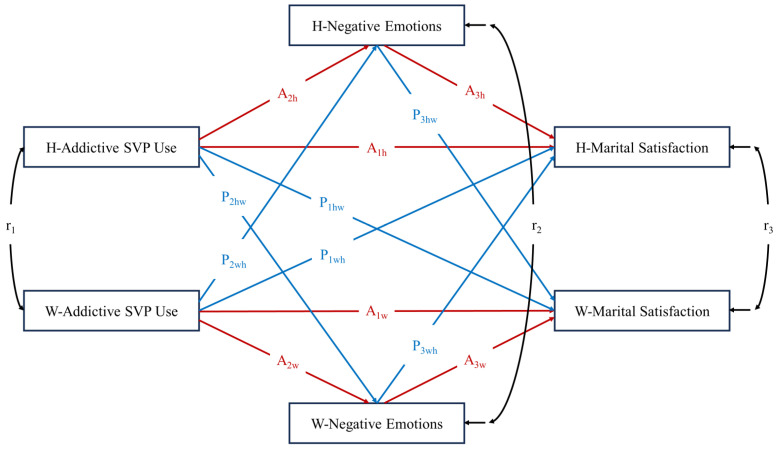
Hypothesized actor–partner interdependence mediation model. *Note*. W = wives; H = husbands. The structure of the model comprises six actor effects (indexed as A, represented by red arrows) and six partner effects (indexed as P, represented by blue arrows). Subscript 1 denotes a direct effect, while Subscripts 2 and 3 represent indirect effects. Additionally, h and w represent the husband and wife, respectively. The notation *hw* signifies the husband’s effect on the wife, and *wh* signifies the wife’s effect on the husband.

A paired sample *t*-test (for continuous variables) and McNemar test (for categorical variables) was employed to assess gender differences in the primary research variables. Pearson correlation was employed to examine the associations between specific demographics (e.g., age and education level) and other research variables, as well as to calculate intercorrelations among the primary research variables. These preliminary analyses were performed using SPSS version 25.0. An APIM [34] was then applied to test the interdependent effects. The APIM takes into account the interdependencies between pairs of data and allows for the statistical modeling of the mutual influence that partners may have on each other. Additionally, an actor–partner interdependence mediation model (APIMeM) [35] was generated to examine the hypothesized mediators. The APIMeM is presented in Figure 1, which estimated the influences of individuals’ addictive SVP use (X_A_) on their own or their partner’s negative emotions (X_M_), as well as their own or their partner’s marital satisfaction (X_Y_). We analyzed the indirect effects and assessed them for statistical significance by checking whether the bootstrapped samples (*k* = 5000 samples) had a 95% confidence interval (CI) that excluded zero. Mplus version 8.3 was used to test the APIM and APIMeM. Three fit indexes were employed to evaluate the fit of the hypothesized models: (1) comparative fit index (CFI), which assessed the fit in comparison to other models and indicated acceptable fit when CFI value exceeded 0.95; (2) root-mean-square error of approximation (RMSEA), which was considered indicative of acceptable fit when its value was lower than 0.06; and (3) standardized root-mean-square residual (SRMR), where a value below 0.08 suggested acceptable fit [36].

## 4. Results

### 4.1. Preliminary Analyses

When examining gender differences, it was observed that the husbands were, overall, older than the wives—*M*_husbands_ = 69.47, *SD*_husbands_ = 8.88 vs. *M*_wives_ = 66.84, *SD*_wives_ = 8.08, *t* (263) = 11.489, *p* < 0.001—and the husbands exhibited a higher level of education than the wives—χ^2^ (9, 264) = 53.399, *p* < 0.001. As presented in Table 1, there were no gender differences in measures of addictive SVP use, negative emotions, or marital satisfaction (see Table 1).

In terms of the bivariate correlations between demographics and the primary research variables, it was found that age showed a negative correlation with addictive SVP use among both husbands (*r* = −0.312, *p* < 0.001) and wives (*r* = −0.276, *p* < 0.001), while education level was not associated with the primary research variables for either group. As there were no statistically significant correlations observed between age or education level and marital satisfaction among neither the husbands nor the wives in this study, we did not include any demographic variables as controls in the subsequent analyses.

Table 2 presents the intercorrelations of responses to the primary research variables. The scores of spouses on each measure exhibited moderate associations, ranging from approximately *r* = 0.40 to *r* = 0.60 These findings indicate a substantial level of agreement in how spouses perceive specific relational experiences.

### 4.2. Actor and Partner Direct Effects: APIM

To test the actor and partner direct effects of addictive SVP use on marital satisfaction, an APIM analysis was conducted. As the model was saturated, meaning that all the estimated parameters precisely matched the elements in the covariance matrix and the degrees of freedom were zero, the estimation of fit indexes was not conducted, and only the path coefficients were deemed relevant in this case [37].

**Figure 2:**
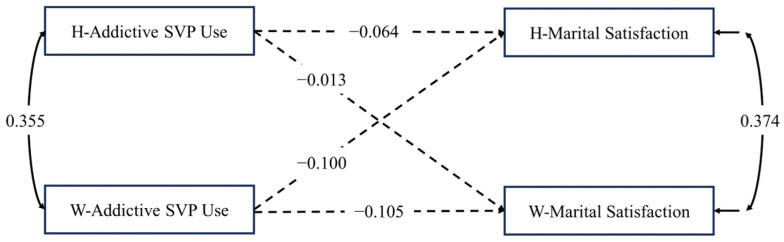
Unstandardized path estimation of addictive SVP use (X_A_) on marital satisfaction (X_Y_). *Note*. W = wives; H = husbands. Dashed lines indicate no statistical significance at *p* = 0.05 level.

Regarding H1, neither the husbands’ nor the wives’ actor direct effects were significant. Regarding H3, neither the husbands’ nor the wives’ partner direct effects were significant (see Figure 2, Table 3).

### 4.3. Actor and Partner Indirect Effects: APIMeM

**Figure 3:**
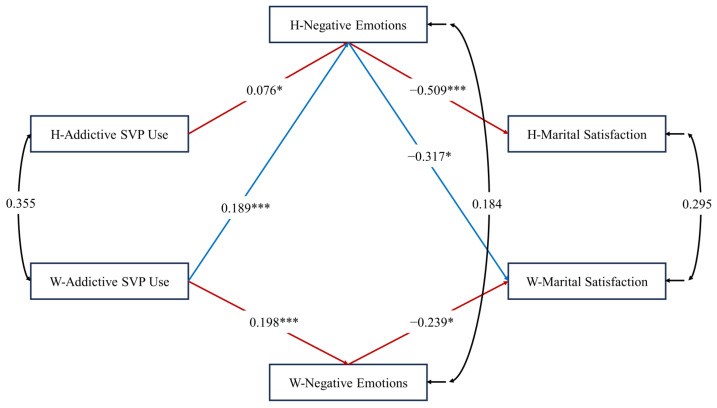
Unstandardized path estimation after removing of non-significant paths. *Note*. W = wife; H = husband. Red arrows represent the actor effect, while blue arrows represent the partner effect. The model fit indexes were as follows: χ^2^(6) = 4.835, *p* = 0.565; comparative fit index = 1.000; root-mean-square error of approximation = 0.000; standardized root-mean-square residual = 0.023. * *p* < 0.05; *** *p* < 0.001 (two-tailed).

To test the actor and partner indirect effects of addictive SVP use on marital satisfaction mediated by negative emotions, an APIMeM analysis was conducted. As shown in Figure 3 and Table 4, we first ran the saturated model. In this model, six paths were found to be non-significant. These paths included the actor effects of both partners’ addictive SVP use on their own marital satisfaction (A1h, A1w), the partner effect of the husband’s addictive SVP use on his wife’s marital satisfaction (P1hw), the partner effect of the wife’s addictive SVP use on her husband’s marital satisfaction (P1wh), the effect of the husband’s addictive SVP use on his wife’s negative emotions (P2hw), and the effect of the wife’s negative emotions regarding the husband’s addictive SVP use on her husband’s marital satisfaction (P3wh). We then removed these six paths. The final APIMeM reflected adequate overall fits: χ^2^(6) = 4.835, *p* = 0.565; CFI = 1.000; RMSEA = 0.000; SRMR = 0.023. Furthermore, upon incorporating the mediator variable, negative emotions, into the model, we observed a reduction in the correlation coefficient for marital satisfaction between wives and husbands from its original value of 0.374 (Figure 2) to 0.295 (Figure 3). This suggests that the mediator variable further elucidates the relationship originally explained by wives’ and husbands’ addictive SVP use.

#### 4.3.1. Actor Indirect Effects

In line with H2, the actor indirect effects were found to be significant in both the husbands and the wives. Specifically, in the husbands’ segment of the model, addictive SVP use was positively related to negative emotions, and then adversely to marital satisfaction (*B* = −0.038, 95% CI = [−0.079, −0.003]). Similarly, the wives’ addictive SVP use was positively related to their own negative emotions, and then adversely to their own marital satisfaction (*B* = −0.047, 95% CI = [−0.102, −0.005]).

#### 4.3.2. Partner Indirect Effects

Partially in line with H4a, the wives’ addictive SVP use was positively associated with their husbands’ negative emotions, and then adversely to their husbands’ marital satisfaction (*B* = −0.096, 95% CI = [−0.159, −0.045]). However, a similar pattern was not observed in the partner–actor indirect effect on wives’ marital satisfaction. Additionally, there was no statistical significance in the actor–partner indirect effect on either the husbands’ or the wives’ marital satisfaction. Therefore, H4b was not supported. Finally, partially in line with H4c, the wives’ addictive SVP use was positively related to their husbands’ negative emotions, which, in turn, was adversely related to the wives’ marital satisfaction (*B* = −0.060, 95% CI = [−0.129, −0.011]). No similar pattern was observed in the partner–partner indirect effect on the husbands’ marital satisfaction, either.

## 5. Discussion

This study sought to explore how addictive SVP use in Chinese older couples is linked to their reduced marital satisfaction, with the mediators of both spouses’ negative emotions playing a role in this connection. APIM with indirect paths was used to test the hypotheses.

### 5.1. Hypothesis Model

Using displacement theory and self-perception theory as our groundwork, we assumed that addictive SVP use in older adults would be negatively related to their own declining marital satisfaction and that their increased negative emotions might be the mediator in addictive SVP use causing emotional issues, thereby resulting in the deterioration in their own marital satisfaction. Contrary to our hypotheses, neither the husbands’ nor the wives’ addictive SVP use was found to be directly related to their own marital satisfaction. However, when considering the mediating effect, the harmful effects of addictive SVP use on marital satisfaction were transmitted by the negative emotions of both husbands and wives. These findings suggest that as long as older adults do not experience negative emotions, their SVP use may not necessarily lead to a decline in their marital satisfaction. It could be that, if they have enough time for entertainment, their excessive SVP use will not interfere too much with other aspects of their life and allow them to still maintain a positive emotional state, as well as meaningful interactions with their spouse. However, if their SVP addiction causes them to feel upset or ashamed, then it is likely that they will also experience a decrease in closeness and less-satisfactory interactions with their partner.

Through the lens of social exchange theory, we speculated that one’s addictive SVP use would lead to a deterioration in their spouse’s marriage satisfaction because the addictive use would reduce meaningful interactions and moments to build connection within their spouse, making the spouse feel less rewarded in the marriage, which, in turn, would lead to emotional unhappiness and less satisfaction with the marriage. Partially in line with our hypotheses, the partner effect was only supported from the perspective of the wife. Husbands perceived more negative emotions towards their wife’s addictive SVP use which, in turn, led to them experiencing a greater decline in marital satisfaction (*B* = −0.096), surpassing the effect of the husband’s own indirect influence (*B* = −0.038). The husband’s negative feelings towards his wife’s SVP addiction also led to a decrease in the marital satisfaction of the wife (*B* = −0.060), exceeding the wife’s own indirect effect (*B* = −0.047). These findings emphasize the substantial impact of the spouse’s addictive SVP use on the dynamics of the relationship (see Table 3). In contrast, the husband’s partner effect was not significant in that their addictive SVP use was not related to their wife’s negative emotions.

There could be two reasons for this unexpected finding. First, the wife’s addictive SVP use could be considered to be a deviation from the Chinese traditional gender division norm in families, which violates the behavioral prescription of an eligible wife and thus makes the husband less satisfied. Chinese traditional gender norms can be described as men being the breadwinners, while women are homemakers, with husbands required to earn a desirable amount of money while wives take on the responsibilities of doing housework and taking care of the family [38]. The respondents in our study were older adults, and had already retired, at which point the husband no longer needs to spend their time being a money-maker. As such, the husband’s addictive SVP use does not violate the behavioral expectations of men, nor does he deviate from the gender division norm. However, even when older, the wife continues to be the home-maker, and addictive SVP use will reduce the time they have to do the housework, which violates the behavioral prescription of being a “good wife” and deviates from the gender norm. When this deviation occurs, the destructive behavior of one partner leads to dissatisfaction in the other spouse [39]. As addictive SVP use seemed to only cause deviations in the wives’ gender norms, the decline in marital satisfaction may therefore occur only for husbands. Second, husbands and wives may differ in how they derive satisfaction from marriage [12], with men tending to regard their wives as the main caretakers responsible for catering to their needs [40]. Additionally, men typically rely more extensively on their spouses within their social networks for emotional support compared to women. In later stages of life, a spouse assumes a pivotal role in shaping the overall wellbeing of older men [41]. As a result, husbands might be more sensitive to their wives’ responsiveness, and when their wives are absorbed in SVPs, the husbands may feel neglected and less satisfied. Future research could potentially incorporate a measure of both spouses’ adherence to traditional roles [42] and their social support to further elucidate this aspect.

### 5.2. Strengths, Limitations, and Future Research Directions

When interpreting the findings of this study, it is important to consider both its strengths and limitations. First, to the best of our knowledge, this study is the first to explore the connection between media addiction and relationship satisfaction among older couples in China. Previous studies in this area have been criticized for focusing too much on Western or undergraduate samples [43]. Second, by taking advantage of the dyadic nature of our data, we were able to look at potential gender differences within couples, rather than simply the differences between isolated male and female participants. Third, by examining negative emotions as the mediator, we provide a possible explanation for the associations between dyads’ addictive SVP use and their marital satisfaction.

This study offers a significant theoretical contribution by not only investigating the impact of addictive use of SVPs on marital satisfaction among older couples but also by discerning the divergent pathways through which this addiction influences older women and men in their relationships. This detailed understanding of the specific pathways enriches our comprehension of technology’s role in shaping interpersonal dynamics, particularly within the context of later-life relationships, and holds particular significance within the framework of Chinese cultural norms and values. Practically, couples should evaluate and manage their SVP use and be open to discussing potential overuse of the platforms to prevent experiencing negative feelings themselves or causing them in their partners. To cultivate healthier use habits and ensure adequate interaction time for healthy marital functioning, older adults could enable inter-application settings within their mobile devices or applications, allowing for the establishment of daily or weekly tracking or limits. By identifying negative emotions as the process through which addictive use may harm marital satisfaction, “emotion-focused” therapy might be a useful approach when treating older individuals or couples exhibiting dissatisfaction in their marriage due to maladaptive SVP use, or who are frustrated by their spouse’s addictive use. One existing intervention program has indicated that media addiction can be effectively mitigated through cognitive reconstruction and supportive techniques [44]. The cognitive reconstruction phase helps individuals become aware of the adverse consequences of media addiction and the potential benefits of reducing their media usage, and reinforcing this awareness further can contribute to a significant reduction in media addiction. Additionally, as algorithms are sometimes used to cause user addiction [45], the executives of SVPs should be held responsible for evaluating and protecting users from the potential harms of addictive use, especially among more vulnerable groups, including children and adolescents [46].

At least two limitations of this study should be noted. First, although we suggest that addictive SVP use is likely to have a negative impact on marital satisfaction, we acknowledge that declined marital satisfaction may cause addictive use. There could be a reciprocal influence between the two, and this cannot be ruled out by the cross-sectional design used in the current study. In fact, a recent study has actually revealed that a decrease in satisfaction in romantic relationships can trigger addictive Instagram use [8]. Other research has suggested that a couple could use online media as their primary coping strategy when faced with relationship struggles, which may point to the possibility that marital dissatisfaction predicts addictive media use [47]. Therefore, we suggest that future research should investigate the potential pathways with a more nuanced design, such as utilizing a diary to capture fluctuations in addictive SVP use and marital satisfaction to delineate causality. Another potential research design involves reducing the use of SVPs in older adults and observing its effects on negative emotions and marital satisfaction. Second, our design used self-reporting data, which are vulnerable to recall bias and social desirability bias. In fact, older adults are especially susceptible to recall bias due to their decline in memory [48,49]. Our use of door-to-door visits may also have led to socially desirable bias in that people may be inclined to understate their SVP usage or negative emotions while overstating their marital satisfaction, as they may have been conscious of the presence of the researcher and/or their spouse, thus making the association studied more salient. Furthermore, this reporting may vary between older men and women [50], potentially contributing to the gender disparities observed in the current study. To reduce self-reporting bias in future research, tracking the objective usage of SVPs with the help of software and inviting the dyadic partners to participate separately should be considered. Moreover, the utilization of physiological indicators, such as heart rate variability, to measure negative emotions, and behavioral interaction metrics between spouses, can offer a more objective assessment of marital satisfaction. Given the increasing attention paid by researchers to the phenomenon of “phubbing” in recent years [51], particularly within the context of intimate relationships such as those between partners [52], parents and children [53], and friends [54], future research could distinguish whether SVP use is primarily engaged in within the partner’s view or not. This differentiation could enhance our understanding of the implications of technology addiction on older couples.

## 6. Conclusions

This study is one of the first to explore the reciprocal effects of addictive media use on marital satisfaction among Chinese older couples. Our findings provide a deeper insight into the impact of new media technologies on the wellbeing of older adults by focusing on the increasingly popular SVPs. We demonstrate how the addictive use of these technologies can generate negative emotions and dissatisfaction within users’ dyadic partnerships. Investigating the mechanisms and outcomes of media use among older adults can help elucidate how new media technologies can either facilitate or impede the process of active aging.

## Figures and Tables

**Table 1:** Gender differences in the primary research variables.

	Husbands (n = 264)		Wives (n = 264)			
	*M*	*SD*	*M*	*SD*	*t* (263)	*p*
Addictive SVP use	1.730	0.937	1.669	0.909	0.989	0.323
Negative emotion	1.298	0.576	1.345	0.650	−1.394	0.165
Marital satisfaction	4.076	0.824	4.060	0.841	0.323	0.747

**Table 2:** Zero-order correlations of spouse responses on the primary research variables.

	1	2	3	4	5	6
1. H—addictive SVP use						
2. H—negative emotions	0.265 ***					
3. H—marital satisfaction	−0.119	−0.356 ***				
4. W—addictive SVP use	**0.419** ***	0.349 ***	−0.140 *			
5. W—negative emotions	0.149 *	**0.594** ***	−0.300 ***	0.277 ***		
6. W—marital satisfaction	−0.062	−0.327 ***	**0.560** ***	−0.119	−0.357 ***	

*Note*. W = wife; H = husband. Associations between spouses on the same variable are indicated by bold values. * *p* < 0.05; *** *p* < 0.001 (two-tailed).

**Table 3:** Unstandardized effects of the actor–partner interdependence model testing the effect of addictive SVP use (X_A_) on marital satisfaction (X_Y_).

Effects	X_A_	X_Y_	*B*	*SE*	*p*
Actor direct					
	H—addictive SVP use	H—marital satisfaction	−0.064	0.061	0.294
	W—addictive SVP use	W—marital satisfaction	−0.105	0.071	0.140
Partner direct					
	H—addictive SVP use	W—marital satisfaction	−0.100	0.061	0.100
	W—addictive SVP use	H—marital satisfaction	−0.013	0.076	0.861

*Note*. W = wife; H = husband.

**Table 4:** Unstandardized effects of the actor–partner interdependence mediation model testing the indirect effect of negative emotions (X_A_) on the association between addictive SVP use (X_M_) and marital satisfaction (X_Y_).

Effects	X_A_	X_M_	X_Y_	*B*	*SE*	Lower 2.5%	Upper 2.5%
Actor indirect							
Actor–actor	H—addictive SVP use	H—negative emotions	H—marital satisfaction	−0.038	0.019	−0.079	−0.003
	W—addictive SVP use	W—negative emotions	W—marital satisfaction	−0.047	0.025	−0.102	−0.005
Partner indirect							
Partner–actor	W—addictive SVP use	H—negative emotions	H—marital satisfaction	−0.096	0.029	−0.159	−0.045
Actor–partner	H—addictive SVP use	H—negative emotions	W—marital satisfaction	−0.024	0.017	−0.068	0.000
Partner–partner	W—addictive SVP use	H—negative emotions	W—marital satisfaction	−0.060	0.030	−0.129	−0.011

*Note*. W = wife; H = husband.

## Data Availability

The raw data supporting the conclusions of this article will be made available by the authors on request.
